# Specialized attachment structure of the fish pathogenic oomycete *Saprolegnia parasitica*

**DOI:** 10.1371/journal.pone.0190361

**Published:** 2018-01-17

**Authors:** Svetlana Rezinciuc, Jose Vladimir Sandoval-Sierra, Yolanda Ruiz-León, Pieter van West, Javier Diéguez-Uribeondo

**Affiliations:** 1 Department of Mycology, Real Jardín Botánico CSIC, Madrid, Spain; 2 UTAI, Real Jardín Botánico, CSIC, Madrid, Spain; 3 International Centre for Aquaculture Research and Development at the University of Aberdeen, Aberdeen Oomycete Laboratory, Institute of Medical Sciences, Foresterhill, Aberdeen, United Kingdom; Agriculture and Agri-Food Canada, CANADA

## Abstract

The secondary cysts of the fish pathogen oomycete *Saprolegnia parasitica* possess bundles of long hooked hairs that are characteristic to this economically important pathogenic species. Few studies have been carried out on elucidating their specific role in the *S*. *parasitica* life cycle and the role they may have in the infection process. We show here their function by employing several strategies that focus on descriptive, developmental and predictive approaches. The strength of attachment of the secondary cysts of this pathogen was compared to other closely related species where bundles of long hooked hairs are absent. We found that the attachment of the *S*. *parasitica* cysts was around three times stronger than that of other species. The time sequence and influence of selected factors on morphology and the number of the bundles of long hooked hairs conducted by scanning electron microscopy study revealed that these are dynamic structures. They are deployed early after encystment, *i*.*e*., within 30 sec of zoospore encystment, and the length, but not the number, of the bundles steadily increased over the encystment period. We also observed that the number and length of the bundles was influenced by the type of substrate and encystment treatment applied, suggesting that these structures can adapt to different substrates (glass or fish scales) and can be modulated by different signals (*i*.*e*., protein media, 50 mM CaCl_2_ concentrations, carbon particles). Immunolocalization studies evidenced the presence of an adhesive extracellular matrix. The bioinformatic analyses of the *S*. *parasitica* secreted proteins showed that there is a high expression of genes encoding domains of putative proteins related to the attachment process and cell adhesion (fibronectin and thrombospondin) coinciding with the deployment stage of the bundles of long hooked hairs formation. This suggests that the bundles are structures that might contribute to the adhesion of the cysts to the host because they are composed of these adhesive proteins and/or by increasing the surface of attachment of this extracellular matrix.

## Introduction

Oomycetes are successful and widespread groups of parasites [1]. In order to colonize and parasitize, they rely on asexual motile zoospores, *i*.*e*., secondary zoospores [2–4] that form secondary cysts that attach to their host prior the infection [5–8]. The ability of the oomycetes to invade a wide range of hosts such as fungi, algae, plants, invertebrates, or vertebrates, indicates that they might have adaptable mechanisms for substrate and host colonization [9–13]. Recent genome and secretome studies have shown that these mechanisms involve diverse molecules including translocated host-targeting proteins [14], degrading enzymes, such as glycosyl-hydrolases and proteases [15], or adhesive biopolymers [16, 17]. These adhesive compounds allow cell-substratum attachment, prevent the pathogen from removal, facilitates host-pathogen interaction and host colonization [18].

Biological attachment is based on adhesive polymers that can vary in structure and capabilities involving interactions and components with different functions [19]. In the plant pathogenic oomycetous species, *Phytophthora cinnamomi*, the adhesion of cysts to plants roots involved the secretion of high molecular weight glycoproteins by rapid exocytosis [11]. In 2005, Robold and Hardham [10], identified a potential adhesive molecule of 220-kDa protein with thrombospondin repeats [10]. In *Saprolegnia parasitica* and *Saprolegnia diclina*, the presence of an extracellular matrix was evidenced by Burr and Beakes [20]. The identification of these components represents a biochemical and genetic challenge since little results have been obtained because of the complexity of the methods and approach [12, 16, 17, 20].

In fungi, similar molecules have been described in *Colletotrichum graminicola* [21], *Erysiphe graminis* [22], *Nectria haematococca* [23]. In the attachment process, some plant pathogenic oomycetes develop specialized attachment structures named appressoria that help the host penetration and subsequent infection [24]. Apart from appressoria, the only structures that have been related to attachment in the oomycetes are the so-called hooked “hairs” or “spines” on the secondary cysts of the fish pathogen *S*. *parasitica* [25]. Theses hooked hairs seem to be unique to the genus *Saprolegnia*, and were described for the first time by Manton et al., [26] using transmission electron microscopy (TEM) and a few years later Meier and Webster [27] reported that *S*. *parasitica* had long bundles of these hooked hairs. All studies on hooked hairs were focused on their morphology and structure and cyst coat ornamentation in *Saprolegnia* spp. [28–35]. Thus, these studies showed that the hooked hairs of *S*. *parasitica* are specifically longer, over 2 μm in length than those of other *Saprolegnia* spp. and are organized in bundles [29, 31]. These hairs appear to be rapidly synthesized during the secondary cyst stage [29]. Beakes [29] presented the overall ontogeny of encystment vesicles in several *S*. *parasitica* isolates. In these vesicles, the hairs seem to be deposited filled with matrix material that seems to be composed of glucose and mannose since it binds to lectin Concavalin A [20]. Later, Beakes et al. [31] suggested that the bundles of long hooked hairs might play a role as a potential virulent factor by increasing the attachment efficiency. Their function is unclear and has been extensively debated in the literature [25]. Thus, it has been suggested that these structures assist the attachment to a substrate [27, 28].

The synchronization of *Saprolegnia* developmental stages *in vitro* [35, 36] as well as the recent sequencing of the whole *S*. *parasitica* genome makes it now possible to investigate secreted protein families with potential roles in virulence at different developmental stages [15]. Therefore, the main objective of this work was to elucidate the function of the bundles of long hooked hairs by: (i) comparing the strength of attachment of the secondary cysts of this pathogen with those of other closely related species that have no long hairs, (ii) understanding the time sequence of external development of these structures, and the influence of the selected factors on the morphology and the number of bundles of long hooked hairs developed, and (iii) finally, by investigating proteins that might be produced during their formation.

## Material and methods

### Strain cultivation, sporulation, and production of cysts

The strain of *S*. *parasitica* (SAP0206) [37] was used in all the experiments. For microscopic studies and assessment of the strength of adhesion of the secondary cysts, the strains SAP0655 of *Saprolegnia delica* [35], and SAP1148 of *Saprolegnia anisospora* [38], from the culture collection of Real Jardín Botánico at CSIC were used. Cultures were maintained on a peptone-glucose agar (PGA) medium [39], and sporulation was performed following the protocol described by Diéguez-Uribeondo et al., [36]. Briefly, mycelia colonies were grown in 0.5 mL peptone glucose liquid media (PG-l) for 24–48 hours at 20°C. The sporulation was induced by washing the mycelia with autoclaved tap water three times and then incubated for 15 hours at 20°C to allow release of the zoospores. The secondary zoospores were collected according to Cerenius and Söderhäll [40] followed by the secondary cysts production through vigorous agitation for 30 sec at 1400 rpm, except of those experiments designed to evaluate the effect of the encystment triggers on the secondary cysts morphology.

### Scanning and transmission electron microscopy

The secondary zoospores were encysted as described above and volumes of 0.5 mL of secondary cysts suspensions were transferred onto glass cover slips (8 mm in diameter). Samples were incubated at 20°C for 70 min and fixed for 1 hour in 2% glutaraldehyde at room temperature. Fixed samples were dehydrated in alcohol series from 30 to 100% and critical point dried. Then, the secondary cysts on selected surfaces were sputter coated in a vacuum with an electrically conductive layer of gold to a thickness of approximately 80 nm. Samples were observed under a Hitachi s3000N scanning electron microscope, SEM, (Real Jardín Botánico, CSIC, Madrid, Spain) at a beam specimen angle of 45°. Accelerating voltage was 20 kV; final aperture was 200 μm.

For transmission electron microscopy, TEM, the samples of secondary cysts obtained as above were processed by using a slight modification of the method described by Beakes [29]. Thus, formvar-coated copper grids (30 mm, 200 mesh) were kept in autoclaved tap water in order to avoid drying, and then fixed in uranyl acetate 2%. The specimens were observed in a JEM-1010, Jeol transmission electron microscope (Centro Nacional de Microscopía Electronica, University of Complutense, Madrid, Spain). Accelerating voltage was 100 kV; resolution 0.3 nm.

### Evaluation of the adhesion strength of the secondary cysts

The adhesion strength of the secondary cysts was tested on selected strains of the species *S*. *anisospora*, *S*. *delica*, and *S*. *parasitica*. Volumes of 0.5 mL of a concentration of 10^4^ secondary cysts/mL were placed onto glass cover slips immediately after encystment. A number of 50 independent suspensions for each species were generated for the experiment. Measurements of the strength of adhesion were performed 70 min after encystment. This allowed secondary cysts to extend their hooked hairs and to attach firmly to the surface. In order to measure the strength required to remove the secondary cysts, we followed the protocol of Letourneau et al., [41]. Briefly, coverslips (8 mm in diameter) with a monolayer of attached secondary cysts were individually vortexed in 1.5 mL Eppendorf tubes for 60 sec at 1400 rpm. The total number of secondary cysts that remained attached was counted before and after the treatment by selecting three arbitrary fields of view and using a light microscope 20x (Olympus BX51, Olympus Optical, Japan). The adhesion strength of the secondary cysts was calculated by first obtaining the average numbers of cysts attached for each replicate (each having measurements of three arbitrary fields) before and after the treatment, and then obtaining the ratio of these averages after and before the treatment.

### Time sequence study of extracellular deployment of bundles of long hooked hairs

The length and number of bundles of long hooked hairs (from now on will be named bundles) produced by the secondary cysts of *S*. *parasitica* were studied in a time sequence series on fixed samples using SEM. Thus, suspensions of secondary cysts obtained as described above were transferred onto glass cover slips (8 mm in diameter). The time series were prepared by vortexing the zoospores for 30 s followed by incubations for 5, 15, 70 and 115 min. After incubations, samples were fixed and processed as described above. For measurements of the length and number of bundles, 25 randomly selected secondary cysts were taken at each treatment tested. The length and number of the bundles was measured for each cyst and the whole number and length of the 25 samples were analyzed for each selected time point. Measurements were performed using ImageJ v.1.51a software [42].

### Effect of substrate, area of contact, and encystment triggers on the length and number of bundles of long hooked hairs

To evaluate the effect of area of contact, and encystment triggers on the number and length of bundles of *S*. *parasitica*, the secondary cysts were obtained as described above and were transferred onto the fish scales (*Salmon salar*). In order to check the effect of an additional surface exposure of the cysts, a suspension of carbon particles was added when the secondary cysts were vortexing. For the experiment, a carbon particle solution was prepared as described in Diéguez-Uribeondo et al., [43]. In the encystment trigger experiments, the secondary cysts were obtained by adding an equal volume of peptone (PG-l), or 100 mM calcium solution (CaCl_2_) to the zoospore suspension with the concentration of 10^4^ zoospore/ml as described by Diéguez-Uribeondo et al., [44].

In all experiments, the suspensions of secondary cysts were incubated for 70 min at 20° C and fixed for SEM as described above. The encystment induced by vortexing followed by sample incubation on glass coverslips was used as control. To count and measure the number and the length of bundles, 25 random SEM micrographs were selected. All counts and measurements were done using ImageJ v.1.51a software [42].

### Enzyme digestions and immunolocalization of bundles of long hooked hairs and extracellular matrix

#### Enzymatic treatments

The following enzyme preparations were tried for digestion of cyst spines: (i) Peptide:N-Glycosidase F (PNGase F, from *Elizabethkingia miricola*, Sigma-Aldrich, Co. LLC, St. Louis, IL) and O-glycosidase (from *Streptococcus pneumoniae*, Sigma-Aldrich, Co. LLC, St. Louis, IL) mix for 2 hours at room temperature with a final enzyme concentration 1.25μ/mL for enzymatic deglycolysation surface proteins; and (ii) Trypsin (from porcine pancreas, Life technologies) for spines dissociation. Trypsin digestion was performed at room temperature for 48 hours with a final enzyme concentration 25mg/mL. Suspensions of cysts *circa* 0,5mL were allowed to lay on glass slides, and were placed onto Petri dishes to prevent evaporation, and incubated for 2 hours followed by fixing step for 1 hour in 4% paraformaldehyde in phosphate buffer 25mM at pH 6. All samples were prepared in two variants on fixed and not fixed cells.

#### Immunolocalization

The cysts were treated with (1) PNGase F only or (2) mix solution of PNGase F and O-glycosidase as described above. Later anti-β-tubulin antibodies, produced in mouse, were applied as described in [45]. Briefly, after enzymatic treatment, samples were permeabilized with 0.1% Triton-X 100 for 15 min and incubated in the presence of monoclonal anti-β-tubulin antibodies (diluted 1: 5000) (Sigma-Aldrich, Co. LLC, St. Louis, IL), produced in mouse for 2 hours followed by a fixing step for 1 hour in 4% paraformaldehyde in PBS. Secondary cysts monolayers were washed carefully three times with PBS, before fixation in 4% paraformaldehyde/PBS for 2 hours at room temperature. The bind of primary antibody was detected using fluorescein isothiocyanate (FITC)-conjugated conjugated goat anti-mouse IgG (H+L). Secondary antibodies were used as controls under identical conditions. Subsequently, the samples were washed three times with phosphate buffer solution (PBS) and incubated with the secondary antibody (goat-anti-rabbit Alexa Fluor 488 conjugate; Invitrogen, No. A31627) according to the manufacturer’s protocol. Samples were washed with PBS three times, and viewed using a Zeiss LSM 510 Meta confocal microscope with a Plan Apochromat x 63/1.0 water-dipping objective lens.

### Bioinformatic analyses

In order to provide the basis for the bioinformatic identification of the potential bundles components, we analyzed the *S*. *parasitica* strain CBS223.65 secretome generated by Broad Institute of MIT and Harvard (https://www.broadinstitute.org) on different life stages, *i*.*e*., growing mycelium, sporulating mycelium, cysts, and germinating cysts(Jiang et al. 2013) To verify this, the predicted secretome proteins of *S*. *parasitica* were analyzed for the presence of signal peptide using SignalP tool (http://www.cbs.dtu.dk/services/SignalP/). Potential secreted proteins were selected based on presence of glycosylation sites by NetNGlyc 4.0 [46] and/or search for conserved catalytic protein domains using ScanProsite [47] database. For functional annotation of the secreted proteins, BLAST tools were used to compare the protein sequences to the NCBI (http://www.ncbi.nlm.nih.gov/).

### Statistical analysis

The data sets were analyzed using a Shapiro-Wilk test to assess the normal distribution of the results. One-way ANOVA analysis was used to compare: (i) the ratio of adhesion strength of secondary cysts among the *Saprolegnia* spp (ii) the length and the number of the bundles of *S*. *parasitica* produced at each time sequence, (iii) and the length and the number of bundles of *S*. *parasitica* produced when secondary cysts were exposed to additional surface and encystment triggers. ANOVA was followed by a Tukey post-hoc test to determinate significant differences among the groups. The ANOVA analyses were carried out in R v. 3.3.0 software [48].

The length of the bundles of the *S*. *parasitica* produced at each time point was evaluated to determinate the corresponding biological development model using four non-linear models, *i*.*e*., Brody, Logistic, Von Bertalanffy, and Gompertz [49]. The best biological development model was found using the easynls R package [50] by comparing multiple non-linear models. To evaluate if the length of bundles is related to the length of anchor points (carbon particle) a linear model was used. The analysis was done using lineal model function of R v. 3.3.0 software [48]. *P*-values <0.05 were considered statistically significant.

## Results

### Scanning and transmission electron microscopy

**Fig 1:**
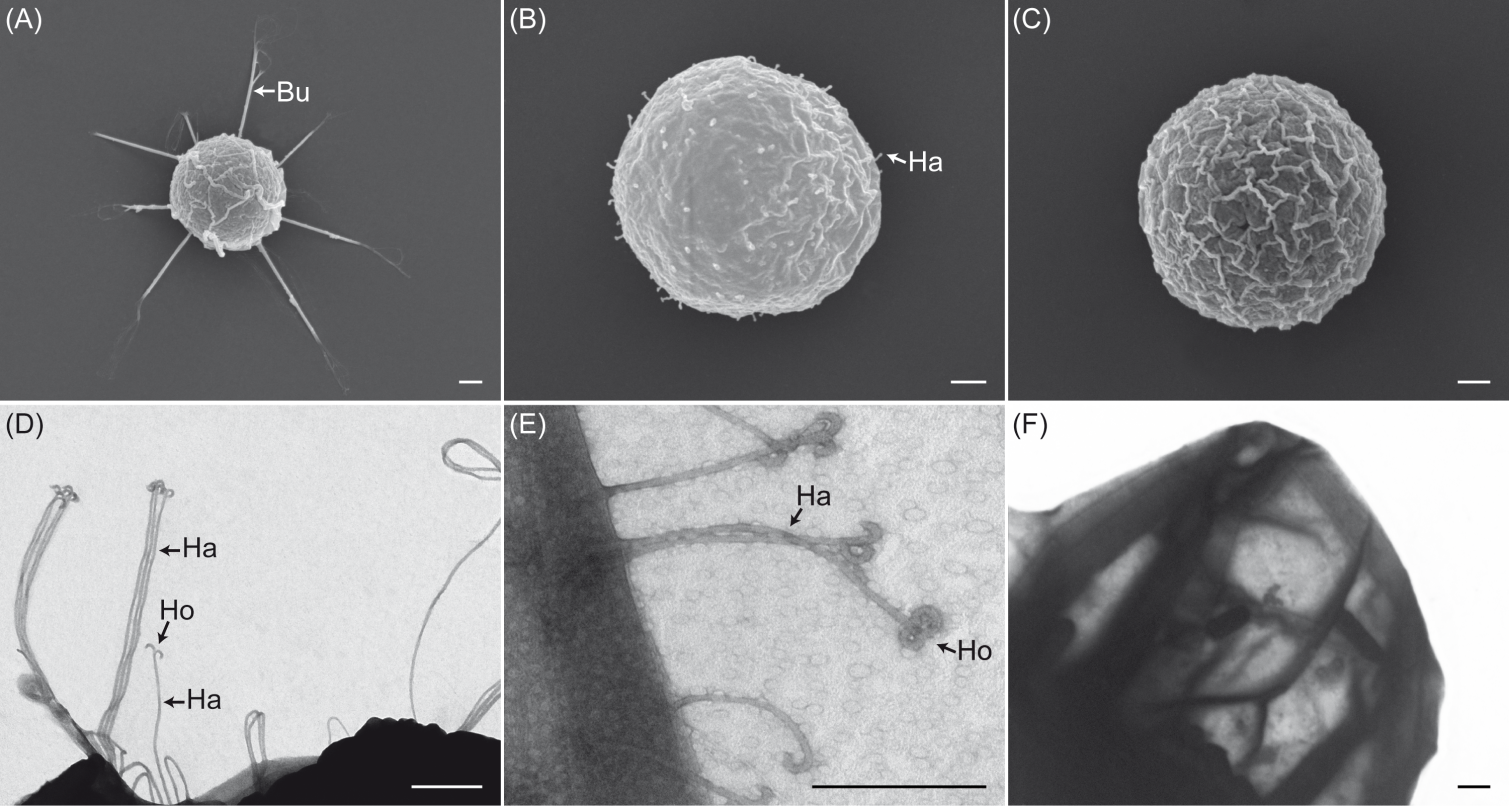
Electron micrographs of secondary cysts and their cell walls from selected *Saprolegnia* species. Scanning electron micrographs of secondary cysts of *S*. *parasitica* (A), *S*. *delica* (B), and *S*. *anisospora* (C) showing presence or absence of bundles (Bu) or single hairs (Ha). Transmission electron micrographs of both *S*. *parasitica* (D), and *S*. *delica* (E) confirmed the presence of hooks (Ho) at their tips and that cysts of *S*. *parasitica* have longer hairs (Ha) than those of *S*. *delica*. Cysts of *S*. *anisospora* do not form any hairs (F). *Scale bar* 1μm.

Scanning and transmission electron microscopy studies on secondary cysts and cell walls of secondary cysts obtained from selected *Saprolegnia* species confirmed that the cysts of *S*. *parasitica* possess bundles of long hooked hairs (10.14±1.40 μm) (Fig 1A and 1D), while *S*. *delica* has single short hooked hairs (0.67±0.09 μm) (Fig 1B and 1E), and *S*. *anisospora* has undecorated cysts (Fig 1C and 1F). Transmission electron micrographs of the hairs of both *S*. *parasitica* and *S*. *delica* showed presence of hooks at their tips (Fig 1D and 1E). In *S*. *parasitica*, these hairs were long and grouped in bundles while in *S*. *delica* the hairs were short and single (Fig 1D and 1E).

### Evaluation of the adhesion strength of the secondary cysts

**Fig 2:**
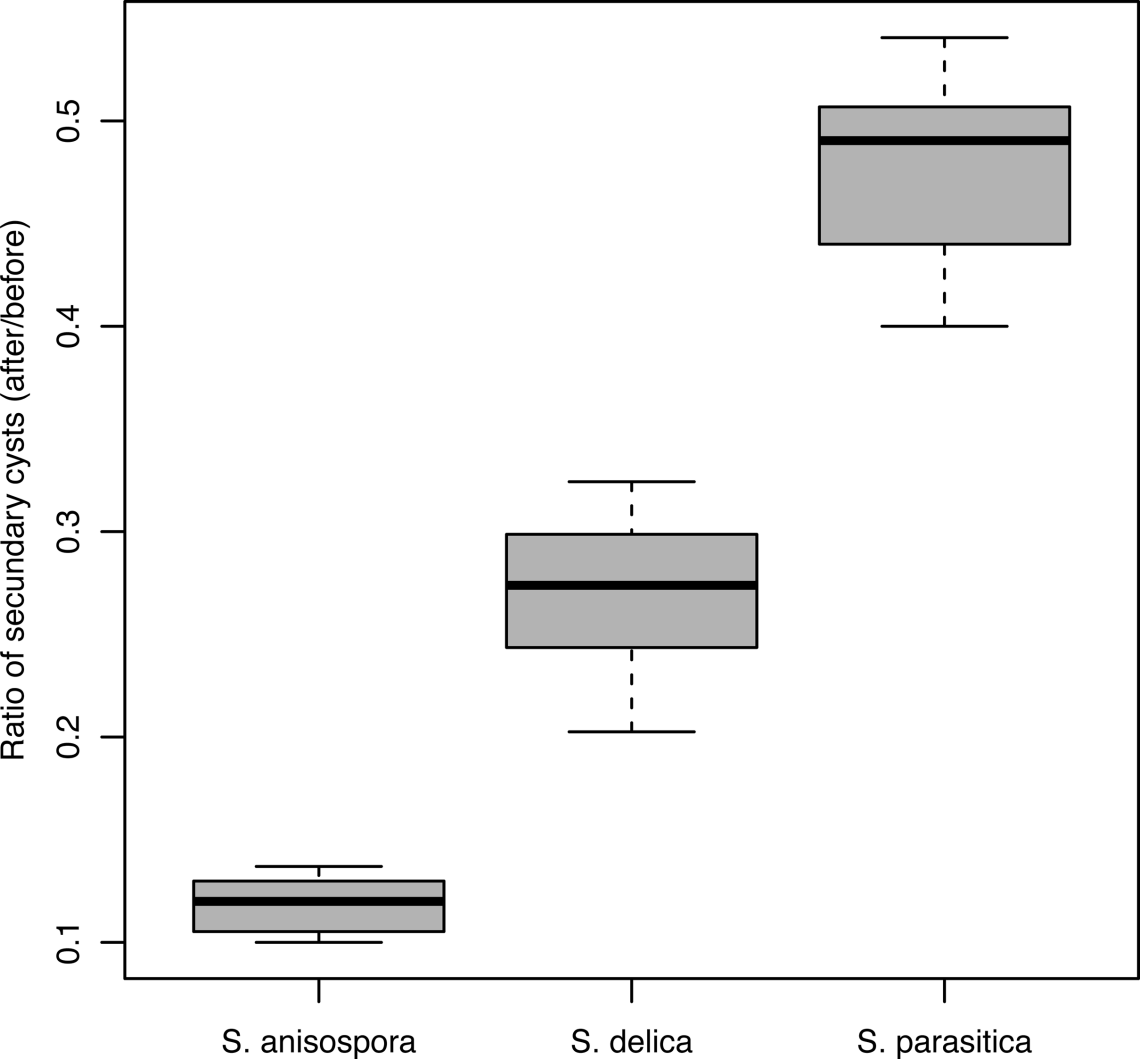
Adhesion strength of the secondary cysts of selected *Saprolegnia* species. The figure shows the ratio secondary cysts attached to glass coverslips after and before treatment in *S*. *parasitica*, *S*. *delica*, and *S*. *anisospora*.

The adhesion strength of the secondary cysts was significantly different for *S*. *parasitica*, *S*. *delica* and *S*. *anisospora* (*F*_2_ = 1776, *p* <2e-16) (Fig 2). The adhesion strength of the secondary cysts was 0.48±0.04 for *S*. *parasitica*, 0.27±0.03 for *S*. *delica*, and 0.12±0.01 for *S*. *anisospora* (Fig 2).

### Time sequence study on extracellular deployment of bundles of long hooked hairs in *S*. *parasitica*

**Fig 3:**
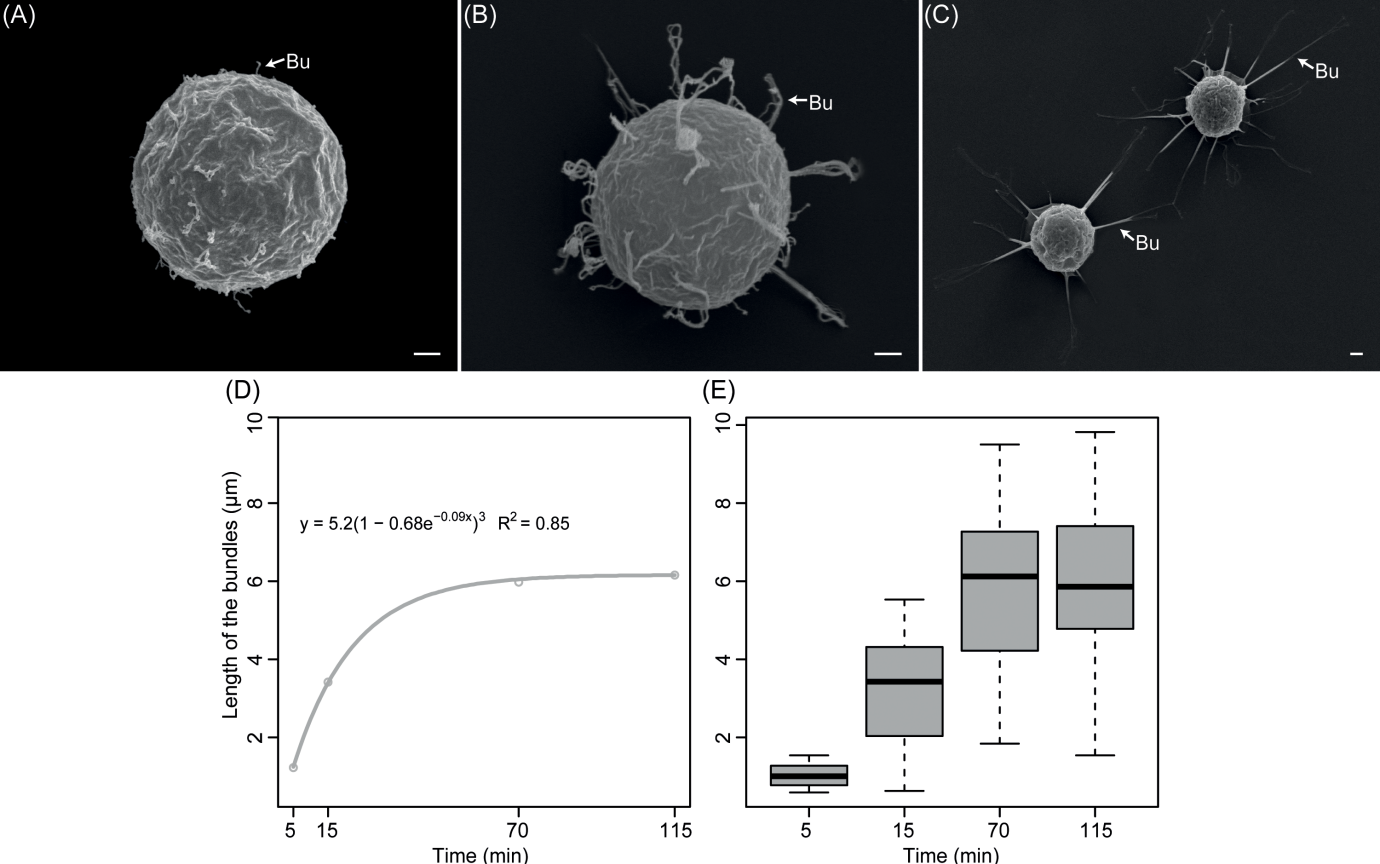
Time sequence of the extracellular deployment of bundles of long hooked hairs on secondary cysts of *Saprolegnia parasitica*. Scanning electron micrographs of the time sequence of the bundles (Bu) of long hooked hairs deployment at 5 (A), 15 (B), and 70 min (C) after encystment (scale bar 1μm). The increase in average length of the bundles of a total of 25 cysts analyzed followed a Brody a non-linear model (D) and was significantly different between selected periods except for periods 70 and 115 min after encystment (E).

The statistical analyses of SEM micrographs of bundles of *S*. *parasitica* revealed that their length varies and increases during the time laps study (Fig 3). Thus, the length was different at time points 5, 15, and 70–115 min (*F*_3_ = 128.90, *p* < 2e-16) (Fig 3D and 3E). At time points 70 min and 115 min, however, the length of the bundles was not significantly different (Fig 3D and 3E). The results indicated that the length of bundles increased immediately after initiation of the encystment, *e*.*g*., 5 min, until about 70 min after encystment following a Brody model (y = a*(1–b*e^(–c*t)^), a = 5.97, b = 1.10, c = 0.06; AIC = 263,30, R^2^ = 0.85) as for the number of bundles at selected time points during encystment revealed no significant differences (12.56±1.59, *F*_3_ = 1.18, *p* = 0.32).

The secondary cysts fixed at 5 min after encystment seemed to have initiated the unfurling of the bundles (Fig 3A). At this time after encystment, the length of bundles was on average of 1.03±0.29 μm (Fig 3E). At time point 15 min after encystment, the bundles increased in length (Fig 3B), and were on average 3.22±1.42 μm (Fig 3E), and in some cases adopted a loop shape (Fig 3B). At 70 and 115 min (Fig 3C), all secondary cysts showed well-extended bundles, and on average, they measured 5.78±2.00 μm, and 5.96±1.98 μm, respectively (Fig 3C and 3E).

### Effect of substrate, area of contact, and encystment triggers on the length and number of bundles of long hooked hairs

**Fig 4:**
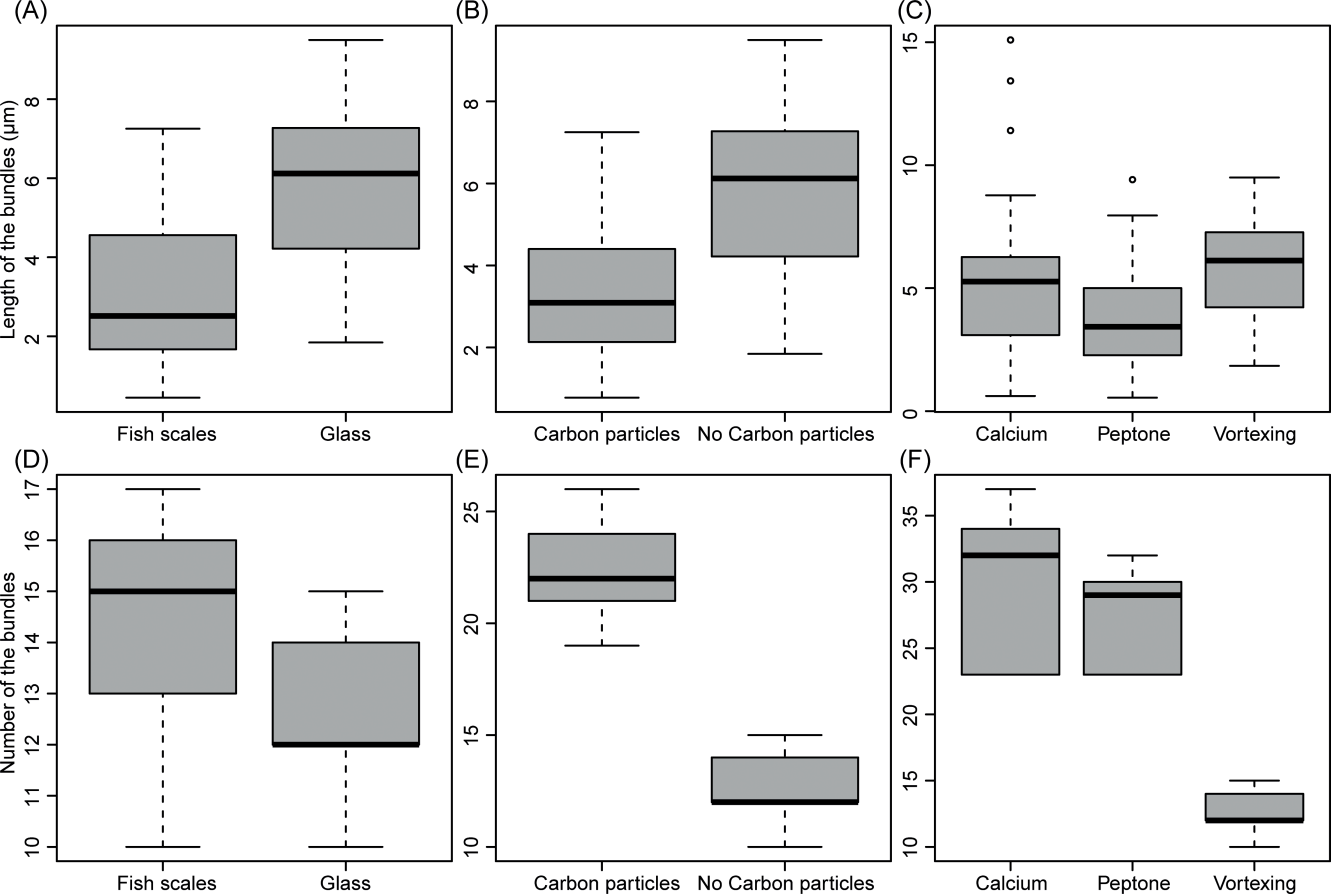
Effect of substrate, area of contact and encystment triggers on the length and number of bundles of long hooked hairs of *Saprolegnia parasitica*. The length of bundles on cysts produced by vortexing and incubated on fish scales was significantly shorter than those incubated on glass slides (A), while the number of bundles was significantly different and higher on fish scales (D). The exposure of secondary cysts to carbon particles resulted in reduction of the length (B) and an increase of the number of bundles (E). The bundles produced by either vortexing or addition of 50 mM CaCl_2_ were significantly longer than those produced by using peptone (3g/l) (C). The number of bundles produced by using calcium or peptone treatments was higher than bundles produced by vortexing (F).

The length and number of the bundles on cysts of *S*. *parasitica* obtained by vortexing and incubated on fish scales were significantly different from those incubated on glass cover slips (Figs 4A and 4D and 6A). The length of the bundles produced on fish scales were on average 3.15±1.87 μm and were shorter than those produced on glass slides (5.78±2.00 μm) (*F*_1_ = 77.39, *p* < 1.8e-15) (Fig 4A). In contrast, the number of bundles significantly increased on the fish scales (14.36±2.22) compared with those incubated on glass slides (12.64±1.47, *F*_*1*_ = 10.47, *p* = 0.002) (Fig 4D).

**Fig 5:**
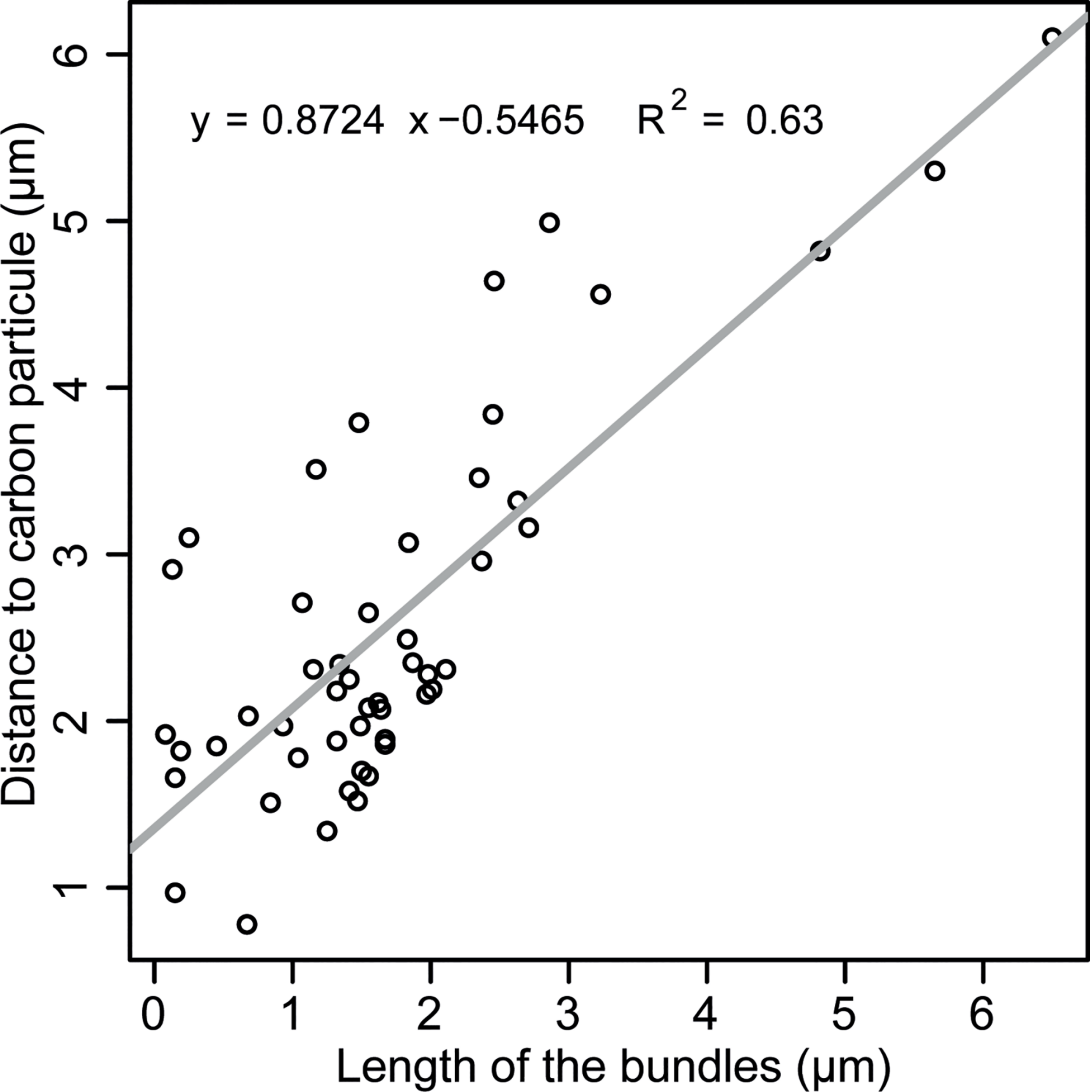
Regression of the length of the bundles of long hooked hairs of *Saprolegnia parasitica* on the distance to anchor points (carbon particles). The figure shows a significant regression of the length (μm) of the bundles produced by the cysts on the distance to carbon particles that were used as anchors points.

**Fig 6:**
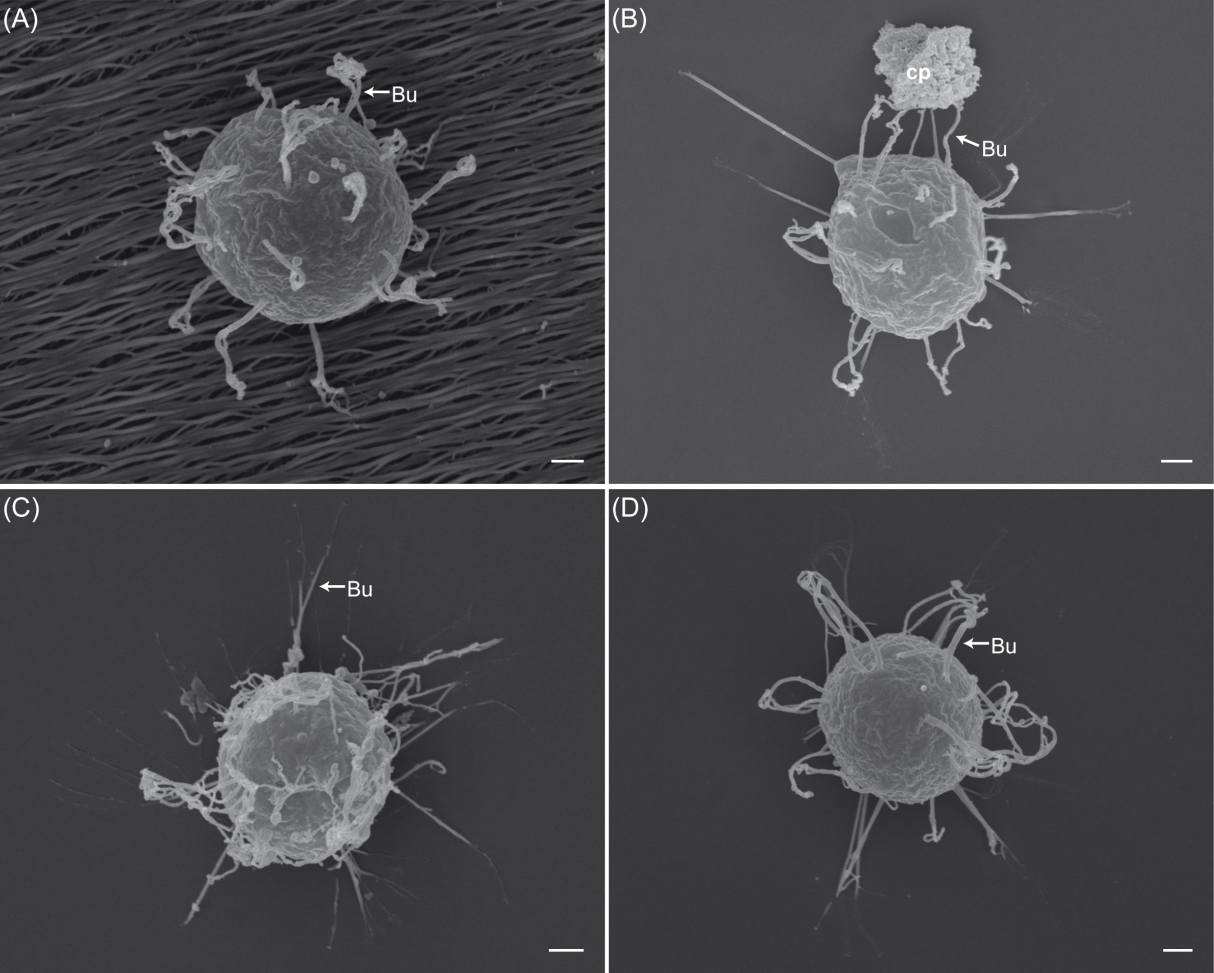
Scanning electron micrographs of secondary cysts of *Saprolegnia parasitica* encysted with selected triggers. Samples of secondary cysts were obtained by: vortexing and incubated on fish scales (A), vortexing with carbon particles (B), incubating in a 50 mM CaCl_2_ on glass cover slips (C), or by incubating in a peptone (3g/l) on glass cover slips (D). The bundles (Bu) of long hooked hairs were shorter on fish scales (A) than those produced by using other treatments (B, C, and D). The number of bundles increased with calcium and peptone treatments (C, D). Carbon particles (cp) seem to represent anchor points for the bundles (Bu). *Scale bar* 1μm.

We found that the exposure of secondary cysts suspensions to an additional surface area, *i*.*e*., carbon particles, resulted in reduction of the length of bundles induced if compared to those produced with no addition of carbon particles (3.35±1.54 μm *versus* 5.78±2.00 μm, *F*_*1*_ = 82.8, *p* <2e-16) (Figs 4B and 6B), while the number of bundles, however, increased (22.68±2.25 *versus* 12.64±1.47, *F*_1_ = 349.2, *p* <2e-1677.39) (Fig 4E). The bundles of cysts produced in this manner, *i*.*e*., carbon particles, were distributed heterogeneously on the surface of the secondary cysts, and we found that there is a correlation between the length of the bundles to the distance from the cyst surface to the carbon particles, *i*.*e*., anchor points (*F*_1,48_ = 81.5, *R*^*2*^ = 0.63, *p* = 6.58e-12) (Fig 5).

Regarding the effect of encystment triggers on length of the bundles, the analysis showed that there are significant differences among encystment triggers (*F*_2_ = 24.75, *p* = 1.09e-10). For example, the average length of the bundles produced by either vortexing (5.78±2.00 μm) or calcium (5.03±2.64 μm) was longer than that produced by using peptone as encystment trigger (3.71±1.87 μm) (*Tukey test*: *p* = 0.063; *Tukey test*: *p* = 2.30e-5, respectively) (Figs 4C and 6C and 6D). However, the average length of the bundles obtained by vortexing or using calcium was not significantly different (*Tukey test*: *p* = 0.063) (Fig 4C).

The analysis of the number of bundles also showed significant differences among encystment triggers (12.64±1.47, *F*_2_ = 141.80, *p* < 2e-16). The number of bundles produced by using calcium (29.48±5.40) or peptone (27.36±3.63) as encystment triggers were significantly different from that produced by vortexing (12.64±1.47) (*Tukey test*: *p* = 0.00; *Tukey test*: *p* = 0.00, respectively) (Fig 4F). However, the number of bundles on cysts of *S*. *parasitica* produced by calcium or peptone was not significantly different (*Tukey test*: *p* = 0.13) (Fig 4F).

### Enzyme digestions and immunolocalization

**Fig 7:**
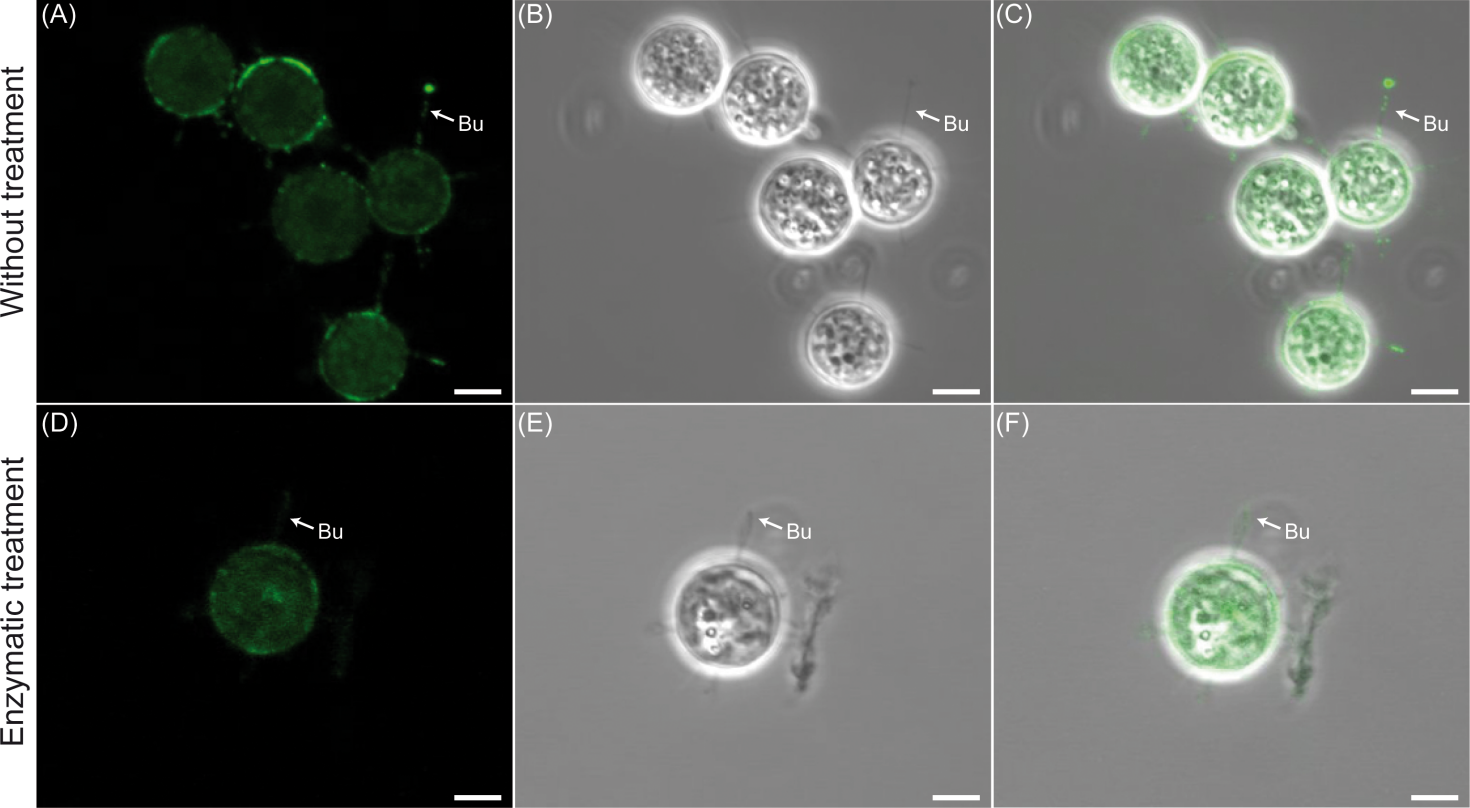
Protein immunolocalization of bundles of hooked hairs of *Saprolegnia parasitica*. The figure shows that treatment with PNGase F prevents the unspecific binding of the anti-β-tubulin antibodies. Differential interference contrast (A and D), confocal light fluorescent (B and E) and merged micrographs (C and F) of bundles (Bu) of hooked hairs of secondary cysts. The cysts were enzymatically treated (D, E, and F) or not (A, B and C) with PNGase F, and incubated in the presence of monoclonal anti-β-tubulin antibodies (1:5000) and goat-anti-rabbit Alexa Fluor 488 conjugate as secondary antibodies (shown in green color). Scale bar 1μm.

The use of histochemical staining was not entirely informative for assessing spines composition due to the presence of *Saprolegnia* adhesive protein complex. This was evidenced by the unspecific binding of the antibodies (Fig 7). Therefore, the digestion with an enzymatic mix consisting of Trypsin, PNGase F, O-glycosidases was applied prior the incubation with antibodies (Fig 7). Digestions with trypsin did not resulted in any rupture of the hairs (Fig 7) and the anti-β-tubulin antibody tested did not bind to the hairs.

### Bioinformatics

The data from the secretome generated by Broad Institute of MIT and Harvard (S1 Table) shows the abundance of approximately 1000 putative secreted proteins from the developmental stages: growing mycelium, sporulating mycelium, cysts, and germinating cysts. We found five putative proteins encoded by the genes SPRG_18636, SPRG_05384, SPRG_19863, SPRG_03974 and SPRG_19320 that might be potential bundle components (Table 1). However, no functional characterization of these or their orthologues have yet been presented. According to the *S*. *parasitica* secretome these five putative proteins are more highly expressed in cysts than in the mycelia (Table 1). SPRG_18636 encodes a 562 amino acid protein, SPRG_05384 encodes a 6547 amino acid protein, SPRG_19863 encodes a 9440 amino acid protein, SPRG_03974 encodes a 5297 amino acid protein and SPRG_19320 encodes a 936 amino acid protein. The predicted catalytic domain of the SPRG_18636, SPRG_05384, SPRG_19863 and SPRG_03974 contain one or more ScanProcite fibronectin III domains, and the SPRG_19320 contains thrombospondin domain (Table 1). The NetNGlyc tool reported positive results for the amino acid sequences of the SPRG_18636, SPRG_05384, SPRG_19863, SPRG_03974 and SPRG_19320 proteins, with 4 to 20 predicted N-glycosylated sites (Table 1).

**Table 1:** List of selected secreted proteins of *Saprolegnia parasitica* with their description and expression levels in different life stages.

Code	PFAM Description	Mycelium	Sporulating mycelium	Cysts	Germinated cysts
SPRG_18636	Fibronectin type III domain	0	0.35	**1.22**	0.30
SPRG_05384	Fibronectin type III domain	0.02	0.09	**8.48**	4.68
SPRG_19863	Fibronectin type III domain	0.04	0.30	**4.60**	1.84
SPRG_03974	Fibronectin type III domain	0.03	6.66	**16.44**	8.30
SPRG_19320	Thrombospondin type 1 domain	0.50	0.42	**227.72**	88.59

## Discussion

The process of attachment is a key host-pathogen interaction mediator and accumulating evidence suggests that pathogenic oomycetes can release specific substances to attach to the host [17, 51, 52]. The involvement in the attachment process of specialized structures is less known. In this study, several approaches have been used to evaluate the attachment strategy of the fish pathogenic oomycete, *S*. *parasitica*. Thus, it has been demonstrated that the bundles of long hooked hairs of *S*. *parasitica* cysts are dynamic structures involved in pathogen attachment.

The strength of cyst attachment of selected *Saprolegnia* spp. appears to be correlated with the length of bundles. Thus, the strength of attachment of *S*. *parasitica* cysts was around three times stronger than that of cysts of two selected *Saprolegnia* spp. that have shorter or lack of hooked hairs. This might indicate that the bundles of long hooked hairs might allow a stronger attachment either by themselves, or by enlarging the area of stickiness [20]. However, it cannot yet be excluded that the more efficient attachment of *S*. *parasitica* cysts might also be the result of different stickiness properties (*i*.*e*., composition) of the cyst extracellular matrix between these three species.

In addition, the evidences found here indicate that these bundles are not passive structures for hooking to the host as proposed by Wood et al., [25]. Instead, they appear to be dynamic attaching structures that are influenced by the characteristics and nature of the attaching surface. We also showed that the deployment of these structures is modulated by both physical and chemical *stimuli* mimicking those occurring at the area of contact with the host. Thus, the bundles of hooked hairs start being arranged extracellularly as soon as 5 min after encystment, suggesting that their components are pre-synthesized before encystment as previously described by Beakes [29]. The bundles increase length but their number remains constant after undergoing either germination or the release of a new zoospore. These two developmental stages are of key importance for the *Saprolegnia* pathogenic species since they determine the successful detection, attachment and colonization, or the formation of a new zoospore [53].

In the plant pathogen *Phytophthora* signals such as Ca^2+^ uptake or mechanical stimulation have been reported to be responsible to induce full and transient fusion of selective secretion of vesicle proteins (which likely to be an important aspect of host infection [13]. In this study, we investigated the effect of both chemical and mechanical *stimuli* on the length and number of bundles. Thus, it was found that these vary depending on substrate, area contact, and encystment triggers. Both the addition of nutrients (*e*.*g*., peptone) or of mechanical *stimuli* (*e*.*g*., carbon particles, rough surfaces) influenced the length and number of the bundles produced. The external formation of the bundles, therefore, might be receptor-mediated and calcium-dependent receptors could be involved. This is evidenced by the fact that addition of high concentration of Ca^2+^ to zoospores increased the number and affected their length throughout the surface of the cyst if compared with mechanical encystment triggers. Burr and Beakes [20], already showed that Ca^2+^ was the only ion that specifically enhanced adhesion of *S*. *parasitica* cyst over *S*. *delica*. Calcium is a non-specific signaling ion that also plays a central role in ion signaling pathways (for review see [54]) and in several important development processes of oomycetes such as encystment, adhesion and germination [55–57]. A variety of specialized structures such as those aiding ingresses of the fungal pathogen into its host, or adsorption of nutrients (appressoria, hyphodamia, haustoria, etc.) are triggered by the presence of certain ions [13, 16, 58, 59]. Furthermore, the dynamic nature of the bundles was evidenced by the fact that their length is also affected by the nature of the surface. Thus, zoospores encysted by agitation and exposed to rough surfaces, such as fish scales, or addition of carbon particles to a surface, formed twisted and curly bundles compared to those on flat surfaces such as glass slides. This might indicate that bundles of hooked hairs external formation could be also the response to mechanical *stimuli*, such as inductive surface cues, or by local accumulation of glue polymerizing agents as it occurs for other oomycetes and also fungi, *i*.*e*., transglutaminases [60].

The immunolocalization studies evidenced the presence of an adhesive extracellular matrix and suggested that β-tubulin is not a component of the bundles. The presence of adhesive matrix on cysts of *S*. *parasitica* was confirmed by enzymatic digestion conducted with both N-glycosidases and O-glycosidases and is an indication of the presence of N-glycosylated glycoproteins covering the surface of the bundles. This finding suggests that this extracellular matrix is composed of N-acetyl-b-glucosaminyl and/or sialic residues as well as N-acetyl-b-d-glucosamineoligomers, which have been shown to be involved in cell attachment of other oomycetes [61].

According to RNA sequence data of the *S*. *parasitica* genome, genes encoding putative secreted proteins [15] are highly expressed in cysts. To verify this, a bioinformatic analysis conducted on secreted proteins specifically expressed at cyst stage [15] has revealed a high expression of putative N-glycosilated proteins with either thrombospondin or fibronectin domains in *S*. *parasitica*. Proteins with these domains are known to be involved in attachment [60]. A similar study conducted on the adhesion of zoospores and cysts to biological and non-biological surfaces by *P*. *cinnamomi* showed importance of glycoproteins in cell binding [12]. The plant pathogen *P*. *cinnamomi* is known to release a glycoprotein cyst coat containing N-acetil galactosamine polysaccharides [17, 62]. Our analyses are unlikely to be sufficient to infer the components of the bundles of hooked hairs but evidence the relationship between the hairs and extracellular matrix, probably, in connection to cell attachment.

Overall, this research provides a number of microscopic, physiological, and bioinformatic evidences supporting the function of the bundles of long hooked hairs as attachment structures of the fish pathogen oomycete, *S*. *parasitica*. These results suggest that these structures are either composed by N-glycosilated proteins or may assist in dispersing the cyst extracellular matrix on the host surface. Further studies should focus on identifying the actual composition of the hairs by for example, selecting secreted proteins by gene disruption or gene silencing [16, 63]. This advance in genomics has opened unprecedented opportunities for *Saprolegnia* investigations. A complete identification of bundles of long hooked hairs compounds and their characterization remains a major research challenge that will help us understand composition of these attachment structures and the manner, in which the adhesion process occurs.

## Supporting information

S1 Table Secretome of *Saprolegnia parasitica*. (TXT)
